# Cortical information flow in Parkinson's disease: a composite network/field model

**DOI:** 10.3389/fncom.2013.00039

**Published:** 2013-04-25

**Authors:** Cliff C. Kerr, Sacha J. Van Albada, Samuel A. Neymotin, George L. Chadderdon, P. A. Robinson, William W. Lytton

**Affiliations:** ^1^Department of Physiology and Pharmacology, State University of New York Downstate Medical CenterBrooklyn, NY, USA; ^2^School of Physics, University of SydneyNSW, Australia; ^3^Brain Dynamics Centre, Westmead Millennium InstituteWestmead, NSW, Australia; ^4^Institute of Neuroscience and Medicine (INM-6) and Institute for Advanced Simulation (IAS-6), Jülich Research Centre and JARAJülich, Germany; ^5^Department of Neurobiology, Yale UniversityNew Haven, CT, USA; ^6^Department of Neurology, Kings County HospitalBrooklyn, NY, USA

**Keywords:** neural field model, spiking neural networks, Parkinsons's disease, thalamus, cortex, basal ganglia, Granger causality, interlaminar processing

## Abstract

The basal ganglia play a crucial role in the execution of movements, as demonstrated by the severe motor deficits that accompany Parkinson's disease (PD). Since motor commands originate in the cortex, an important question is how the basal ganglia influence cortical information flow, and how this influence becomes pathological in PD. To explore this, we developed a composite neuronal network/neural field model. The network model consisted of 4950 spiking neurons, divided into 15 excitatory and inhibitory cell populations in the thalamus and cortex. The field model consisted of the cortex, thalamus, striatum, subthalamic nucleus, and globus pallidus. Both models have been separately validated in previous work. Three field models were used: one with basal ganglia parameters based on data from healthy individuals, one based on data from individuals with PD, and one purely thalamocortical model. Spikes generated by these field models were then used to drive the network model. Compared to the network driven by the healthy model, the PD-driven network had lower firing rates, a shift in spectral power toward lower frequencies, and higher probability of bursting; each of these findings is consistent with empirical data on PD. In the healthy model, we found strong Granger causality between cortical layers in the beta and low gamma frequency bands, but this causality was largely absent in the PD model. In particular, the reduction in Granger causality from the main “input” layer of the cortex (layer 4) to the main “output” layer (layer 5) was pronounced. This may account for symptoms of PD that seem to reflect deficits in information flow, such as bradykinesia. In general, these results demonstrate that the brain's large-scale oscillatory environment, represented here by the field model, strongly influences the information processing that occurs within its subnetworks. Hence, it may be preferable to drive spiking network models with physiologically realistic inputs rather than pure white noise.

## 1. Introduction

**Figure 1:**
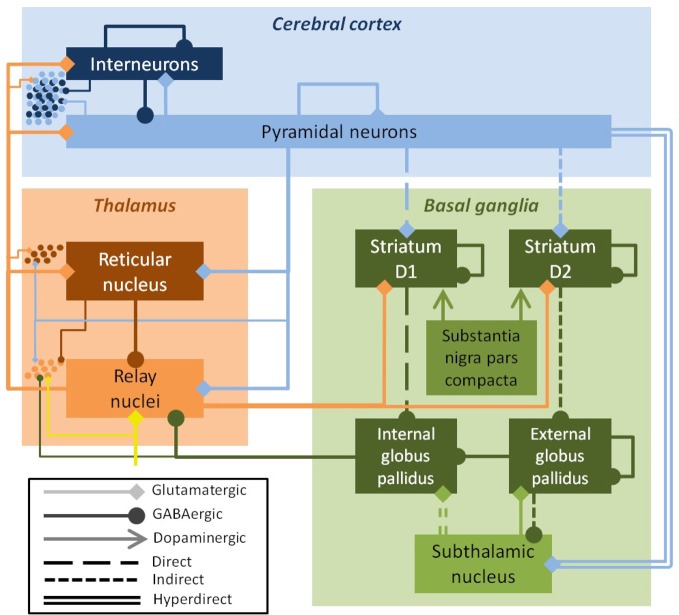
**Schematic of the field model, showing excitatory populations and connections (light colors, diamond arrows) and inhibitory ones (dark colors, round arrows).** The key efferent nucleus of the basal ganglia is the internal globus pallidus (GPi), which receives cortical input via direct, indirect, and hyperdirect pathways. The field model drives a spiking network model, shown here schematically (dots at left); the inputs from the field model to the spiking model are indicated by the thin lines. The substantia nigra pars compacta modulates parameters, but is not explicitly modeled. Inputs to the thalamus (yellow arrow) were modeled as white noise.

Parkinson's disease (PD) is a multiscale phenomenon, encompassing pathology at the level of single neurons, local networks, large neuronal ganglia, and the complex interactions between these ganglia and the cortex. PD is caused by the degeneration of dopaminergic neurons in the substantia nigra pars compacta, with the damage later spreading to dopaminergic neurons in the ventral tegmental area (Cools, 2006). The loss of dopaminergic input alters the dynamics of the striatum, which then affects the dynamics of large portions of the thalamus and cortex, which in turn affects the spinal cord and muscles (Bolam et al., 2002). Striatal dynamics are crucial to several large-scale projection pathways, including the well-characterized direct and indirect pathways. Dopaminergic input to the striatum increases transmission in D1-expressing striatal neurons involved in the direct pathway. These neurons inhibit the globus pallidus internal segment (GPi). Dopaminergic input also decreases input to D2-expressing striatal neurons involved in the indirect pathway. These neurons inhibit the globus pallidus external segment (GPe), which in turn inhibits the GPi. Thus, alterations to the direct and indirect pathways in PD are both thought to increase the firing rate of the GPi, which in turn inhibits the thalamus. There is also a hyperdirect pathway from the cortex to the GPi via the subthalamic nucleus (STN), as well as other lesser pathways (Figure 1).

Numerous models of PD and the basal ganglia have been proposed, using either field or network approaches. Van Albada and Robinson (2009) developed a field-based model of the basal ganglia/thalamocortical system. This model was shown to reproduce realistic firing rates of each neuronal population in both healthy and PD states. One early network model was that of Terman et al. (2002), which represented a small network of neurons in the GPe and STN. A considerably larger and more complex (non-spiking) network model was developed by Leblois et al. (2006). This model explored both basal ganglia and thalamocortical cell populations, looking at competition between the direct and hyperdirect pathways. They suggested that PD disrupted this competitive balance, resulting in loss of the network's ability to select motor programs. Another network model focusing on motor-selection abilities was developed by Humphries et al. (2006), who also found that decreased dopamine interfered with the basal ganglia's capacity for selecting actions. Network models have also been used to analyze and predict the effects of deep brain stimulation on basal ganglia nuclei (Hahn and McIntyre, 2010; Guo and Rubin, 2011; Dovzhenok et al., 2013).

Previous neuronal network models of PD have either not included a cortex at all (Terman et al., 2002; Rubchinsky et al., 2003; Park et al., 2011), approximated it as a random Poisson process (Humphries et al., 2006), or considered it as a single layer with a single cell type (Leblois et al., 2006). The thalamus has also either been omitted or treated as a single population. In this work, we sought to fill this gap by exploring the interactions of the large-scale dynamics of basal ganglia, represented by a field model, with a far smaller but more spatially detailed network model of the thalamus and six-layered cortex.

### 1.1. Composite model

The primary aim of this paper is to determine how the large-scale dynamics of the brain affect the information flow in small networks of neurons. Most previous brain modeling efforts have been directed at one of these two scales, rather than their interaction. These efforts have consisted of either (1) neural field models that describe the dynamics of the whole brain, without explicitly modeling the activity of individual neurons (Nunez, 1974; Jirsa and Haken, 1996; Robinson et al., 1997; Destexhe and Sejnowski, 2009), or (2) spiking neuronal network models that capture individual neurons' dynamics, but are many orders of magnitude smaller than the brains of even the simplest vertebrates (Lumer et al., 1997; Neymotin et al., 2011b). Several large network models have also been published that have roughly as many “neurons” as the full mammalian brain (Izhikevich and Edelman, 2008; Ananthanarayanan et al., 2009). However, these models have not yet reproduced large-scale dynamics with the same degree of fidelity as neural field models. For example, the model of Izhikevich and Edelman (2008) showed simultaneous peaks in the delta and alpha bands, whereas experimentally these peaks are characteristic of sleep and wakefulness, respectively, and are hence rarely observed simultaneously (Niedermeyer and Lopes da Silva, 1999). Such infidelity may be because the enormous computational resources required to run these models makes it impractical to constrain their parameters by fitting their dynamics to experimental data.

Recently, both Deco and Jirsa (2012) and Wilson et al. (2012) described approximations that allow small and large spatial scales to be spanned at a mesoscopic level of description, allowing large-scale dynamics (e.g., BOLD signals) to be related to small-scale network properties (e.g., criticality). Robinson and Kim (2012) took a different approach: they described the theoretical basis of combining spiking network and neural field components into a single model. The fundamental challenge in combining these two modeling approaches is to create a common representation of neuronal activity, since individual spikes are used in network models, while field models use average firing rates. Converting individual spikes into an average firing rate is a straightforward reduction of dimensionality: one simply needs to average over multiple neurons in the model. In contrast, converting an average population firing rate into individual spikes in multiple neurons requires an increase in dimensionality. This is a degenerate problem, so additional assumptions must be made. One approach, described in Robinson and Kim (2012), is to treat each neuron as a phase oscillator. The average firing rate then represents the instantaneous rate of phase change, with a given neuron firing whenever its phase advances by 2π radians. However, here we used an alternative approach, in which the average firing rate is taken as the instantaneous rate for an ensemble of Poisson processes. These are then used to generate individual spike times (Dayan and Abbott, 2001; Leblois et al., 2006; Chadderdon et al., 2012). This approach produces variability in spike timings even with a constant average firing rate, as is seen in real neuronal populations.

## 2. Methods

The model we used consisted of a network of spiking neurons that was “embedded” in a neural field model. The embedding consisted of having the field model generate spikes (via an ensemble of Poisson processes) that were used to drive the network model. Except where otherwise noted, all analyses were performed on the network model. The complete model is publicly available via ModelDB: https://senselab.med.yale.edu/modeldb/ShowModel.asp?model=147366.

### 2.1. Neural field model

The neural field model was based on the work of Van Albada and Robinson (2009) and Van Albada et al. (2009). The neuronal populations and connections that constitute this model are shown in Figures 1 and 3A respectively. The basal ganglia nuclei modeled were the striatum, internal and external pallidal segments, and STN. The internal pallidal population can be thought of as including the substantia nigra pars reticulata, which has very similar connections and properties. The substantia nigra pars compacta was not explicitly modeled, except through its effects on the other nuclei. The thalamus was modeled as two populations: the inhibitory thalamic reticular nucleus (TRN) and the excitatory thalamocortical relay nuclei (TCR). The cortex was also modeled as two populations, representing inhibitory interneurons and excitatory pyramidal neurons. Since together these neuronal populations comprise a large portion of the brain, a network formulation would be computationally intractable. Except for a unitless normalization constant, all parameter values were based on anatomical and physiological data, as listed in Table 2 of Van Albada and Robinson (2009).

In neural field models, neuronal properties are spatially averaged. The dynamics are then governed by a set of equations relating the mean firing rates of populations of neurons to changes in mean cell-body potential, which are in turn triggered by mean rates of incoming spikes. The neural field model used here was based on a previously published model of the electrophysiology of the thalamocortical system (Robinson et al., 1997, 2001, 2002, 2005; Rennie et al., 1999), which in turn was based on earlier field models (Wilson and Cowan, 1973; Nunez, 1974; Freeman, 1975; Steriade et al., 1990; Wright and Liley, 1996).

The first component of the model is the description of the average response of populations of neurons to changes in mean cell-body potential. The mean firing rate *Q*_*a*_ of each population *a* is the maximum attainable firing rate *Q*^max^_*a*_ times the proportion of neurons with a membrane potential *V*_*a*_ above the mean threshold potential θ_*a*_. This can be approximated by the sigmoid function

(1) $$Q_{a}(r,\;t)=\frac{Q_{a}^{\text{max}}}{1+e^{-[V_{a}(r,\;t)-\theta_{a}]/\sigma^{\prime }}},$$

where **r** is the spatial coordinate, *t* is time, and σ′ is $\sqrt{3}/\pi$ times the standard deviation of the distribution of firing thresholds (Wright and Liley, 1995). This function increases smoothly from 0 to *Q*^max^_*a*_ as *V*_*a*_ changes from −∞ to ∞.

The change in the mean cell-body potential due to afferent activity depends on the mean number of synapses *N*_*ab*_ from neurons of population *b* to neurons of population *a* (note that the direction of projection *b* → *a* follows the conventions of control theory and matrix multiplication). The change in potential also depends on *s*_*ab*_, the time-integrated change in cell-body potential per incoming spike. Defining ν_*ab*_ = *N*_*ab*_*s*_*ab*_, the change in the mean cell-body potential in neurons of population *a* is (Robinson et al., 2004).

(2) $$D_{\alpha \beta }(t)V_{a}(t)=\sum_{b}\nu_{ab}\phi_{b}(t-\tau_{ab}),$$

(3) $$D_{\alpha \beta }(t)=\frac{1}{\alpha \beta }\frac{d^{2}}{dt^{2}}+\left(\frac{1}{\alpha }+\frac{1}{\beta }\right)\frac{d}{dt}+1.$$

Here, ϕ_*b*_(*t* − τ_*ab*_) is the incoming firing rate, τ_*ab*_ represents the axonal time delay for signals traveling from population *b* to population *a* neurons, and α and β are the decay and rise rates of mean cell-body potential. The differential operator *D*_αβ_(*t*) represents dendritic and synaptic integration of incoming signals (Robinson et al., 1997; Rennie et al., 2000). The synapses and dendrites form an effective low-pass filter with a cut-off frequency between 1/α and 1/β.

In this model, neuronal activity spreads along the cortex in a wavelike fashion. This reflects previous models (Nunez, 1995; Jirsa and Haken, 1996; Bressloff, 2001) as well as experimental observations of such waves following cortical stimulation (Burns, 1951; Nunez, 1974; Rubino et al., 2006). Estimates of characteristic axonal ranges and propagation speeds suggest that these waves are significantly damped on the scale of the human cortex (Robinson et al., 2001, 2004; Wright and Liley, 1995). Assuming that the range distribution of corticocortical fibers decays exponentially at large distances, activity propagates according to a 2D damped-wave equation of the form (Robinson et al., 1997)

(4) $$Q_{a}(r,\;t)=\left[\frac{1}{\gamma_{a}^{2}}\frac{\partial^{2}}{\partial t^{2}}+\frac{2}{\gamma_{a}}\frac{\partial }{\partial t}+1-r_{a}^{2}\nabla^{2}\right]\phi_{a}(r,\;t),$$

where γ_*a*_ = *v*_*a*_/r_*a*_ is the damping rate, consisting of the average axonal transmission speed *v*_*a*_ (≃10 m·s^−1^) and the characteristic axonal range *r*_*a*_. In practice, most types of axons are short enough to justify setting γ_*a*_ = ∞, which has been termed the local interaction approximation (Robinson et al., 2004). We therefore take only γ_*e*_, the damping rate of cortical pyramidal neurons, to be finite. This turns all wave equations except the cortical one into delayed one-to-one mappings. The model was implemented on a 5 × 5 grid of nodes with coupling to nearest-neighbor nodes via this damped-wave equation.

### 2.2. Spiking network model

**Figure 2:**
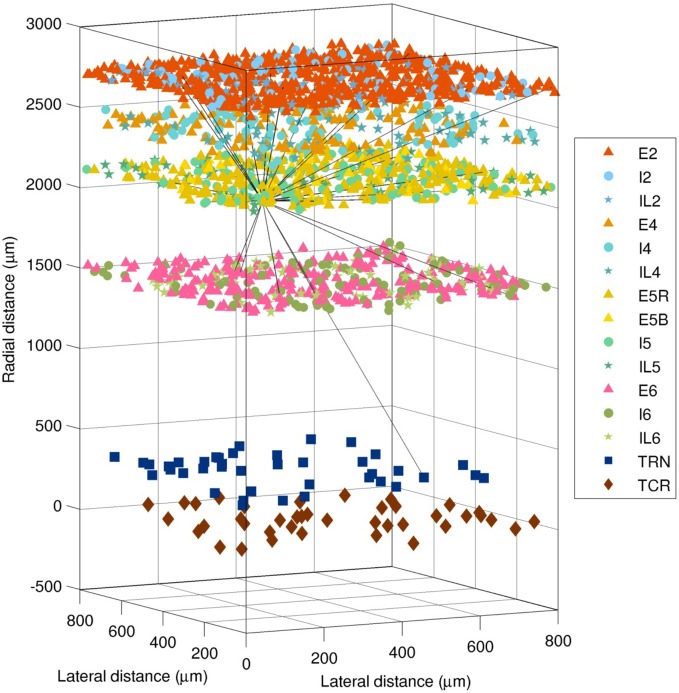
**Layout of the 4950 neurons in the spiking network model (1980 cells shown).** Shapes show type (triangle = excitatory pyramidal, E; circle = fast-spiking interneuron, I; star = low-threshold spiking interneuron, IL; square = thalamic reticular, TRN; diamond = thalamocortical relay, TCR). The 28 efferent connections from a single layer 5 pyramidal neuron are shown (black lines). The distance from the thalamus to the cortex is not shown to scale.

The spiking network was based on several previous models developed by our group (Lytton and Stewart, 2005; Lytton et al., 2008b; Neymotin et al., 2011b; Kerr et al., 2012; Song et al., 2013). It consisted of 4950 event-driven integrate-and-fire neurons. These were divided into three types (excitatory pyramidal cells *E*, fast-spiking inhibitory interneurons *I*, and low-threshold spiking interneurons *IL*), which were in turn distributed across the six layers of the cortex, plus two thalamic cell populations (excitatory thalamocortical relay *TCR* and inhibitory thalamic reticular *TRN*), for 15 distinct neuronal populations in total. The numbers and locations of each neuronal population are illustrated in Figure 2, and were as follows: E2 (i.e., excitatory pyramidal neurons of layer 2/3), 1500; I2, 250; IL2, 150; E4, 300; I4, 200; IL4, 150; E5R, 650; E5B, 150; I5, 250; IL5, 150; E6, 600; I6, 250; IL6, 150; TCR, 100; and TRN, 100. The pyramidal neurons in layer 5 are divided into two populations, *R* (regular firing) and *B* (bursting), since these have different cellular properties and connectivity patterns.

**Figure 3:**
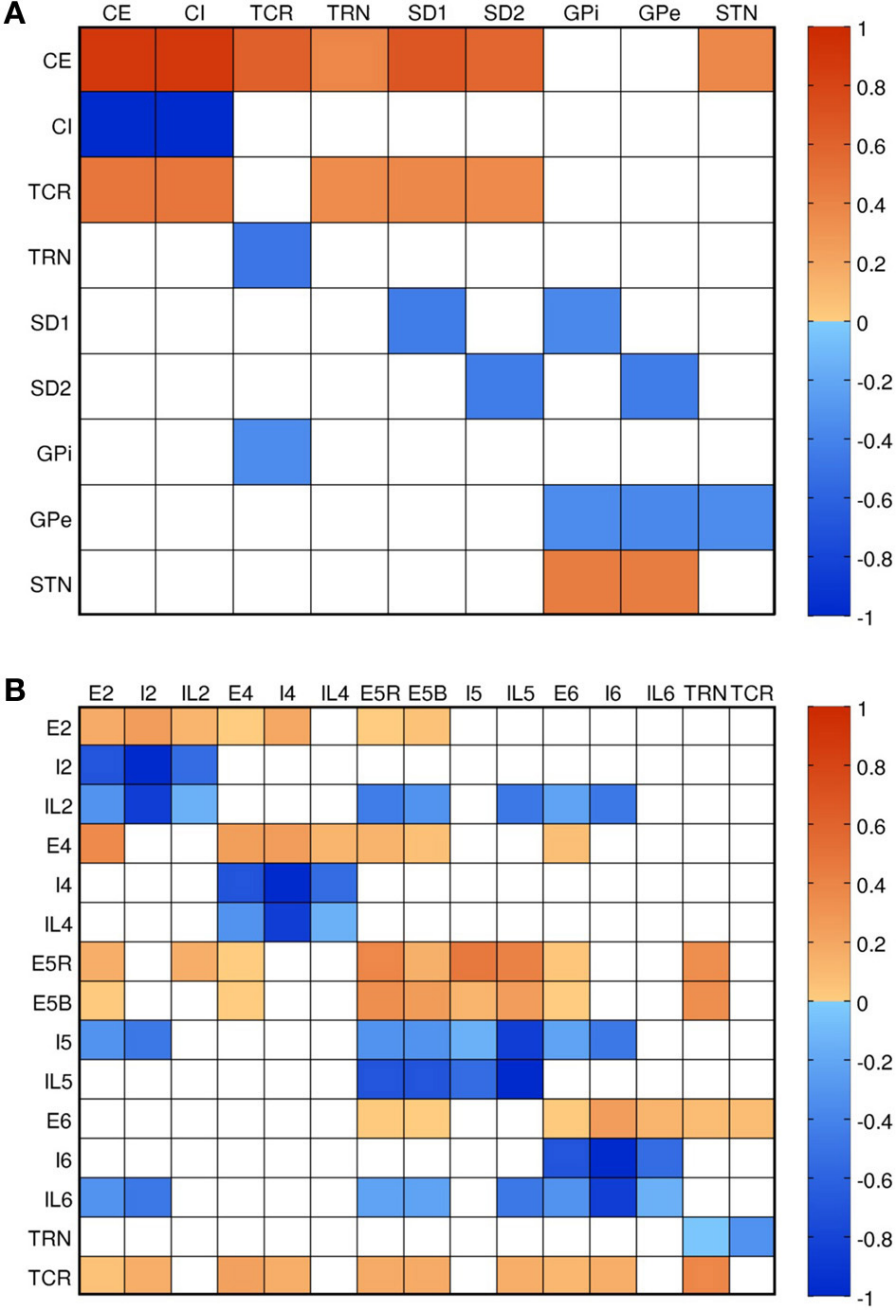
**Connectivity of the models.** Color shows normalized effective connectivity (probability × weight) from rows to columns, with red denoting excitation and blue denoting inhibition. **(A)** Connections in the field model (CE, cortical excitatory; CI, cortical inhibitory; TCR, thalamocortical relay; TRN, thalamic reticular nucleus; SD1, striatal D1; SD2, striatal D2; GPi, internal globus pallidus; GPe, external globus pallidus; STN, subthalamic nucleus). **(B)** Connections in the network model. Approximate diagonal symmetry shows that most connections are reciprocal; relatively strong connections along the diagonal indicate high intralaminar connectivity.

Connectivity (shown in Figure 3B) and the relative numbers of neurons per layer were based on published models (Traub et al., 2005; Neymotin et al., 2011a,b) and anatomical studies (Thomson et al., 2002; Binzegger et al., 2004; Song et al., 2005; Lefort et al., 2009; Adesnik and Scanziani, 2010). Connectivity was strongest between populations within a given layer, as seen from the four clusters visible along the diagonal of Figure 3B. Overall, excitatory neurons had more projections than inhibitory ones, but inhibitory projections were typically stronger. This balanced excitation and inhibition such that the overall gain of the system (the number of additional output spikes for every additional input spike) was close to unity. Such balance is necessary for avoiding the stable but undesirable states of seizure (pathologically high firing) and quiescence (pathologically low firing).

Individual neurons were modeled as event-driven, rule-based units. Since computing resources are finite, a tradeoff must be made between the complexity of neurons vs. the complexity of the network. The neuron model used was complex enough to replicate key features found in real neurons, including adaptation, bursting, depolarization blockade, and voltage-sensitive NMDA conductance (Lytton and Stewart, 2005, 2006; Lytton and Omurtag, 2007; Lytton et al., 2008a,b; Neymotin et al., 2011b), yet was simple enough to connect into large (10^3^ − 10^6^ neuron) networks.

Each neuron had a membrane voltage state variable (*V*_*m*_) with a baseline value determined by a resting membrane potential parameter (*V*_RMP_, set at −65 mV for pyramidal neurons and low-threshold-spiking interneurons, and at −63 mV for fast-spiking interneurons). This membrane voltage was updated by one of three events: synaptic input, threshold spike generation, and refractory period. These events are described briefly below; further detail can be found in the papers and code cited above.

#### 2.2.1. Synaptic input

The response of the membrane voltage to synaptic input was modeled as an instantaneous rise and exponential decay: $V_{n}(t)=V_{n}(t_{0})+w_{s}(1-V_{n}(t_{0})/E_{i})e^{-\frac{t-t_{0}}{\tau_{i}}}$, where *V*_*n*_ is the membrane voltage of neuron *n*; *t*_0_ is the synaptic event time (i.e., *t* − *t*_0_ is the time since the event); *w*_*s*_ is the weight of synaptic connection *s*; *E*_*i*_ is the reversal potential of ion channel *i*, relative to resting membrane potential (where *i* = AMPA, NMDA, or GABA_A_; and *E*_AMPA_ = 65 mV, *E*_NMDA_ = 90 mV, and *E*_GABA_A__ = −15 mV); and τ_*i*_ is the receptor time constant for ion channel *i* (where τ_AMPA_ = 20 ms; τ_NMDA_ = 30 ms; and τ_GABA_A__ = 10 or 20 ms for somatic and dendritic GABA_A_, respectively).

#### 2.2.2. Action potentials

A neuron fires an action potential at time *t* if *V*_*n*_(*t*) > *T*_*n*_(*t*) and *V*_*n*_(*t*) < *B*_*n*_, where *V*_*n*_, *T*_*n*_, and *B*_*n*_ are the membrane voltage, threshold voltage (−40 mV for pyramidal neurons and fast-spiking interneurons, −47 mV for low-threshold-spiking interneurons), and blockade voltage (−10 mV for interneurons and −25 mV for pyramidal neurons), respectively, for neuron *n*. Action potentials arrive at target neurons at time *t*_2_ = *t*_1_ + *l*(*n*_1_, *n*_2_)/*v* + τ_*s*_, where *t*_1_ is the time the first neuron fired, τ_*s*_ is the delay due to synaptic conduction effects, *l*(*n*_1_, *n*_2_) is the axon length between neurons *n*_1_ and *n*_2_, and *v* is the axonal conduction velocity (≃1 m·s^−1^, which is smaller than in the field model, since long-range fibers tend to be more heavily myelinated).

#### 2.2.3. Refractory period

After firing, a neuron cannot fire during the absolute refractory period, τ_*A*_ (10 ms for interneurons and 50 ms for pyramidal neurons). Firing is reduced during the relative refractory period by two effects: first, an increase in threshold potential, $T_{n}(t)=\left(1+Re^{-\frac{t-t_{0}}{\tau_{R}}}\right)\;T_{n}(t_{0})$, where *R* is the fractional increase in threshold voltage due to the relative refractory period (0.25 for interneurons and 0.75 for pyramidal neurons) and τ_*R*_ is its time constant (1.5 ms for interneurons and 8 ms for pyramidal neurons); and second, by hyperpolarization, $V_{n}(t)=V_{n}(t_{0})-He^{-\frac{t-t_{0}}{\tau_{H}}}$, where *H* is the amount of hyperpolarization (0.5 mV for interneurons and 1 mV for pyramidal neurons) and τ_*H*_ is its time constant (50 ms for interneurons and 400 ms for pyramidal neurons).

Local field potentials (LFPs) were computed for each cortical layer as the average membrane voltage across all neurons in that layer; after baseline removal and normalization, this approach is roughly equivalent to summing over all synaptic currents (Mazzoni et al., 2010). While this approach does not take into consideration synaptic and dendritic geometry, this is not possible in the event-driven point-neuron model used here.

Simulations were run in NEURON 7.3 (Hines and Carnevale, 2001; Carnevale and Hines, 2006) on a Linux workstation with an Intel Xeon 2.7 GHz CPU; each 20 s simulation took approximately 10 min to run on a single core. To avoid edge effects, the first and last 2 s of simulated data were discarded. All analyses were performed on the remaining 16 s of simulated data. Since the model is at steady-state and does not incorporate plasticity effects, longer runs produced similar results (data not shown). Model parameters were tuned manually (within physiological limits) to match experimentally observed firing rates, dynamics, and information-theoretic properties, as described in Song et al. (2013).

### 2.3. Input drive

The composite model consisted of the spiking network model being driven by (“embedded in”) the activity of the field model. Since the field model represents a brain region much larger than the network model, the field causally influences the network, but not vice versa. The key methodological novelty of this work is that the spiking network model is thus embedded in an environment with physiologically realistic dynamics (as provided by the field model), rather than the white noise environment such models are typically embedded in.

To obtain realistic firing rates in the network model, the input spiking rate each neuron receives must be bounded. Hence, the firing rate from each neuronal population in the field model was normalized so that the minimum and maximum input spiking rates were 225 and 1125 s^−1^ for excitatory neurons and 30% lower for inhibitory neurons. The input drive was obtained by treating each of these normalized instantaneous firing rates as the rate of an ensemble of Poisson processes for generating spikes. These spikes were then used to drive each population of spiking neurons, using the same connections as used in the field model itself (e.g., excitatory cortical neurons in the network model received input from the excitatory cortical field, the inhibitory cortical field, and the thalamic field); relative connection weights were also set to match those of the field model. Thus, each neuron belonging to a given population in the network model receives the same average rate of input from the field model, but from a separate Poisson process, thereby avoiding artificial correlations in input spike times between neurons.

Four different inputs were explored in this work. First, all neurons in the network were driven by spikes drawn from a spectrally white distribution (“WN”, the white noise model). This represents the control condition, and is identical to the approach used in previous work with the network model (Neymotin et al., 2011b). Second, neurons were driven by the thalamocortical version of the field model (“TC”, the thalamocortical model); i.e., connection strengths to and from the basal ganglia neuronal populations were set to zero. Third, neurons were driven by the full basal ganglia/thalamocortical model described above (“BG”, the healthy basal ganglia model). Finally, neurons were driven by the full basal ganglia/thalamocortical model, using parameter values shown by Van Albada and Robinson (2009) to best match the electrophysiological changes associated with the degeneration of dopaminergic projections to the striatum (“PD”, the Parkinson's disease model).

### 2.4. Analysis

#### 2.4.1. Fano factor

The Fano factor, a common measure of spiking variability (Churchland et al., 2010), is the ratio of the variance to the mean of the spike rate:

(5) $$F=\frac{\sigma^{2}}{\mu },$$

where σ is the standard deviation and μ is the mean of the time series of binned spiking activity across all neurons. To explore spiking variability on a range of different time scales, the time bin size was varied from 1 ms (resulting in 16,000 bins, with an average of roughly 10 spikes per bin) to 8 s (resulting in 2 bins, with roughly 80,000 spikes per bin).

#### 2.4.2. Population burst probability

A population burst (Benayoun et al., 2010) was defined as ≥2 neurons firing within a given 10 ms time bin. The probability of a burst of size *N* was defined as the number of time bins with *N* cells firing divided by the total number of time bins. The relative burst probability was calculated by dividing the observed number of bursts of each size by the number of bursts of that size expected from uncorrelated activity, which in turn was determined via the observed firing rate (averaged over the entire simulation) and the binomial probability distribution.

#### 2.4.3. Spectral granger causality

Information flow was quantified in terms of spectral Granger causality, also called the directed transfer function (Kaminski et al., 2001). Although many alternative tools for inferring causality exist, such as directed transfer entropy (Lizier et al., 2011), no others allow the spectral properties of the signals to be analyzed in detail.

As in standard Granger causality analysis, spectral Granger causality of α(*f*) → β(*f*) is non-zero if prior knowledge of variable α at frequency *f* reduces error in the prediction of β at frequency *f*. The directionality of the causation arises from the fact that Granger causality quantifies how much the history of time series α can be used to predict the future of time series β: if α has a strong causal influence on β, then the prediction error will be reduced.

Spectral Granger causality is calculated by Fourier transforming the multivariate autoregressive model used in standard Granger causality. Hence, the spectral Granger causality from time series α(*t*) to time series β(*t*) is defined as (Cui et al., 2008)

(6) $$G_{\alpha \to \beta }(f)=-\log\left(1-\frac{\left(N_{\alpha \alpha }-\frac{N_{\beta \alpha }^{2}}{N_{\alpha \alpha }}\right)|H_{\beta ,\;\alpha }(f){|}^{2}}{S_{\beta ,\;\beta }(f)}\right),$$

where *N* is the noise covariance, *H*(*f*) is the transfer function, and *S*(*f*) the spectral matrix, as derived from the bivariate autoregressive model of α(*t*) and β(*t*). This analysis was performed in Matlab 2012a using code based on the BSMART toolbox, available via http://www.brain-smart.org.

## 3. Results

The neural field model results were similar to those reported previously (Van Albada and Robinson, 2009; Van Albada et al., 2009), and are briefly presented here for completeness. We then present the overall dynamics of the spiking network model (Kerr et al., 2012), comparing its dynamics for each of the four drives (white noise, the thalamocortical model, the healthy basal ganglia model, and the PD model). Finally, we focus more closely on the alterations that occur in the PD-driven model and their implications. We have split the results into these sections in order to better accomplish our dual goals of (1) presenting the new composite model, and (2) applying this model to help understand the pathophysiology of PD.

### 3.1. Field model dynamics

Firing rates in each neuronal population were similar to those reported previously (Van Albada and Robinson, 2009; Van Albada et al., 2009). Because the drive from the field model to the network model was normalized to a range that provided realistic firing rates in the latter, tonic firing rates had negligible effect on the simulations.

**Figure 4:**
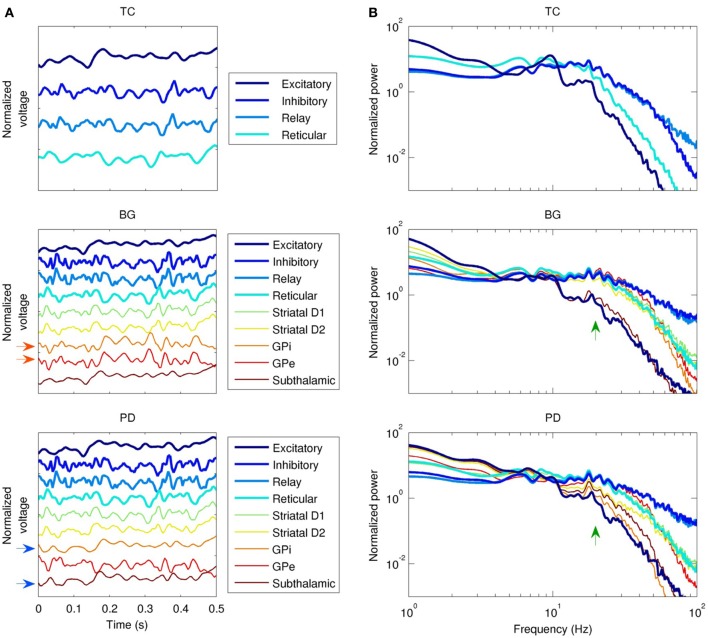
**Dynamics of the three field models (without the network model).** TC, thalamocortical field model; BG, healthy basal ganglia model; PD, Parkinson's disease model; white noise model not shown. “Excitatory” and “inhibitory” refer to cortical subpopulations. **(A)** Local field potential (LFP) time series, showing phase relationships between populations. Activity in the globus pallidus internal (GPi) and external (GPe) segments is normally in phase (red arrows), but this relationship is lost in PD, since the GPi entrains to the subthalamic nucleus instead (blue arrows). **(B)** LFP spectra. Except for the subthalamic nucleus, healthy basal ganglia nuclei spectra are similar to the spectrum of the thalamic relay nuclei from 10–40 Hz. This is disrupted in PD (green arrows), especially in the GPi.

Changes in coherence are a commonly reported finding in PD. In the PD model, coherence between the GPe and the GPi was lost, and high frequency power (>10 Hz) in the GPi was reduced (Figure 4). In the healthy state, activity in the GPe and GPi is strongly correlated (*r*^2^ = 0.9). Following dopamine loss, this correlation is substantially reduced (*r*^2^ = 0.3). This is because the GPe and GPi are both mostly influenced by the striatum in the healthy state, whereas the GPi is strongly driven by the STN in the parkinsonian state, resulting in strong coherence between the GPi and STN in the PD model. Increased STN-GPi coherence at frequencies up to about 35 Hz has indeed been found in PD off levodopa compared to the on-levodopa condition (Brown et al., 2001). Since the GPi is the only nucleus of the basal ganglia that projects to the thalamus or cortex (Figure 1), all changes observed in the network model in the healthy versus PD cases are due to the altered dynamics of the GPi.

To characterize the overall dynamics of the different field models, we looked at their power spectra. In the absence of the basal ganglia, cortical excitatory neurons had a strong alpha peak (10 Hz), and a weaker harmonic in the beta range (20 Hz), as shown in Figure 4B. Cortical inhibitory neurons were driven strongly by thalamocortical cells, evident both in the phase locking between the two populations (Figure 4A), and in the similarity of their power spectra below 70 Hz (Figure 4B). The addition of the basal ganglia (Figure 4B, middle panel) reduced the strength of the alpha peak in cortical excitatory neurons and reduced the slope of the power law spectral fall-off at high frequencies; in cortical excitatory neurons, this slope changed from *P*(*f*) ∝ *f*^−5.3^ to *P*(*f*) ∝ *f*^−4.3^. Reduced dopamine corresponding to PD reduced the power of higher frequencies (>10 Hz) relative to lower frequencies (<10 Hz) in the cortical, thalamic, and GPi spectra. For example, the GPi showed a 2% decrease in power at 10 Hz compared to a 76% decrease at 20 Hz. In contrast, reduced dopamine increased power in the STN at frequencies >10 Hz (e.g., 2.2 times larger at 20 Hz), a result also reported experimentally (Brown et al., 2001; Cassidy et al., 2002; Priori et al., 2004).

### 3.2. Network model dynamics

**Figure 5:**
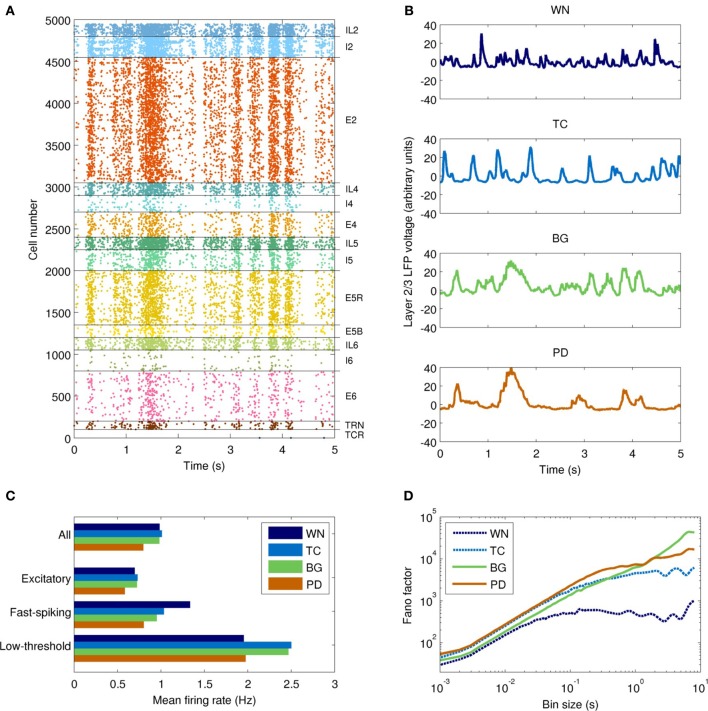
**Temporal dynamics of the network model with each type of input drive (WN, white noise; TC, thalamocortical; BG, healthy thalamocortical/basal ganglia; PD, Parkinson's disease). (A)** Example spike raster from the BG-driven model. Low-frequency oscillations are clearly visible. **(B)** LFPs from layer 2/3 of each model. The BG case corresponds to the raster shown in **(A)**; peaks in voltage are correlated with peaks in spiking activity. **(C)** Mean firing rates by cell type (averaged over both cortical and thalamic populations). Overall, the PD-driven model had considerably lower firing rates, which result from excessive inhibition of the thalamic nuclei. **(D)** Variability in neuronal firing rates on different time scales. The PD- and BG-driven models (which receive the most highly structured input) show the most variability on short and long time scales, respectively; the WN-driven model (which receives the least structured input) shows the least variability on all scales.

The field drive into the network model strongly modulated its spiking activity (Figure 5). Firing rates varied from near zero during the troughs of input activity to >10 Hz during the peaks (Figure 5A). The temporal structure of the spiking activity depended strongly on the type of input drive used (Figure 5B). As a control, white noise produced no consistent temporal structure. The TC-driven model input produced some structure, with a characteristic time scale below 500 ms. The BG-driven model added some features on longer time scales (of order 1 s) to the activity produced by the TC-only field model. Variability in firing rate, as measured by the Fano factor, was lowest in the WN-driven model (Figure 5D)—as would be expected since the white noise had the lowest variability of the four inputs. On time scales <1 s, the PD-driven model had the greatest variability, while the BG-driven model had the greatest variability on scales >1 s.

**Figure 6:**
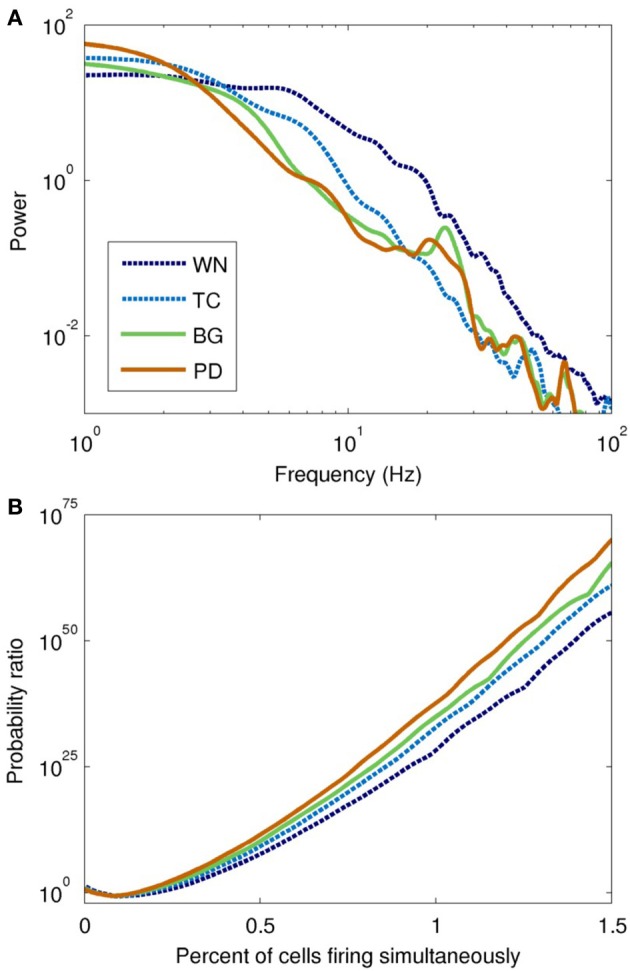
**Spectral and information-theoretic characteristics of the network model as driven by each field model. (A)** Power spectra. The WN- and TC-driven models have fairly featureless spectra, but with different fall-off characteristics at high frequencies. BG- and PD-driven models are similar to the TC-driven spectrum, except for the pronounced peak at ~20 Hz. Spectral power is slightly shifted toward lower frequencies in PD. **(B)** Population burst frequency, defined as the probability of a given number of cells firing within a 10 ms time window, divided by the corresponding probability for uncorrelated processes. All models are many orders of magnitude more likely to show large bursts than would be predicted from uncorrelated activity; large population bursts are most likely in the PD-driven model.

The power spectra of the network model, shown in Figure 6A, were broadly similar to those of the input drives, but with several interesting differences. The basic filter properties of the network model are apparent from the shape of spectrum of the WN-driven model; to a first approximation, the network acts like a low-pass filter, with *P*(*f*) ∝ *f*^−4.0^ for *f* > 20 Hz. However, actual afferent activity in the brain is already low-pass filtered due to dendritic properties, so a more realistic input (the thalamocortical drive) results in even greater low-pass filtering. For example, the WN-driven model predicts 5.4 times more power at 10 Hz than the TC-driven model. Both BG- and PD-driven models differed markedly from the TC-driven model in the 20–30 Hz band, where many basal ganglia nuclei have their peak power. Interestingly, this peak was much sharper in the network model than in the input drive, demonstrating a resonance effect (compare Figure 4A with Figure 6B).

To quantify synchrony in the model on a population level, we used population burst size (Benayoun et al., 2010). All of the field-driven models showed substantially higher population bursting than the WN-driven model (Figure 6B). This is because the field drive applies a global modulatory signal to the network, which organizes the firing of its neurons into up and down states (as evident from the bands of spikes in Figure 5A); in contrast, the WN-driven model has a constant, intermediate level of activation.

### 3.3. Dynamical changes in the parkinson's disease model

The Parkinson's disease model (PD-driven model) showed a number of changes that suggest possible mechanisms underlying the clinical dysfunctions of the disease. Compared to the healthy control (BG-driven model), the PD-driven model showed a shift in the LFP spectrum toward lower frequencies, with higher delta power and a lower beta peak frequency (Figure 6), consistent with clinical findings (Stoffers et al., 2007). These changes were also readily apparent looking at the LFP time series, which showed a flattening of activity between the slow, high-amplitude features (Figure 5B). Soikkeli et al. (1991) noted such slowing in 10 out of 18 non-demented PD patients, as well as in all 18 demented PD patients studied [see Figure 1 in Soikkeli et al. (1991)].

The PD-driven model showed an 18±2% decrease in firing rates compared to the healthy model (Figure 5C), consistent with changes in fMRI indicators of activity (Monchi et al., 2007). The PD-driven model also showed greater firing variability than the healthy model on most time scales. For example, with a bin size of 1 ms, the Fano factor was 41% higher in the PD-driven model (Figure 5D). However, it showed less variability on very long time scales: with a bin size of 8 s, the Fano factor was 2.4 times higher in the BG-driven model. The increased variability in the PD-driven model on all but the longest timescales is consistent with the enhanced oscillations and synchrony associated with PD (Goldberg et al., 2002). Note that maximal dynamical richness does not necessarily correspond to maximal variability in firing rates: for example, tonic firing will have low dynamical richness and low variability on all time scales, while strong, seizure-like oscillations will also have low dynamical richness, despite very high variability (at least on the time scale of the oscillation).

The concentration of activity in large population bursts was a prominent feature of the PD-driven model. For example, bursts consisting of 40 neurons were 60% more common in the PD-driven model than in the healthy model, while 70-neuron bursts were three orders of magnitude more common. (Population bursts smaller than 30 neurons were more common in the healthy model, a result of its higher firing rate.) Although it is tempting to consider these large population events in the context of parkinsonian tremor, we did not note a clear periodicity in their occurrence.

A crucial question in PD is the mechanism by which information flow is disrupted from higher cortical areas (e.g., those involved in motor planning) to primary areas (e.g., those involved in motor execution). Although information flow between cortical layers is bidirectional, a dominant direction of information flow is suggested by both anatomical and functional studies (Bollimunta et al., 2008). This dominant information pathway is believed to stream from thalamic inputs to layer 4 (or upper layer 5 in agranular motor cortices), up to layer 2/3 for processing, and thence to layer 5, which in turn produces outputs to multiple sites including the thalamus, basal ganglia, and brainstem. We hypothesized that damage to this dominant pathway would represent a pathology with major functional consequences. We therefore used Granger causality to quantify information flow between the cortical layers that comprise this pathway.

**Figure 7:**
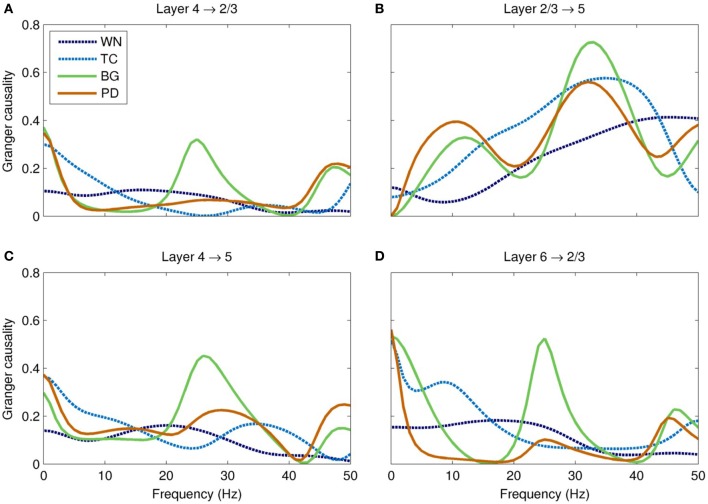
**Spectral Granger causality between cortical layers in each of the models. (A)** The BG-driven model shows strong causality from layer 4 to 2/3 in the delta (<5 Hz) and high-beta/low-gamma (20–35 Hz) bands; causality in the latter band is almost entirely lost in Parkinson's disease. **(B)** The causality from layer 2/3 to layer 5 is slightly reduced in this band in Parkinson's disease. **(C)** These two effects combine to significantly reduce the total Granger causality from layer 4 to layer 5 in PD, especially in the high-beta/low-gamma band. **(D)** Similar reductions of Granger causality in this band were seen in other layer pairs, such as layer 6 to layer 2/3. In each case, the high-beta/low-gamma band Granger causality is significantly higher in the BG-driven model than in any of the other models.

Overall, interlaminar spectral Granger causality was highest in the BG-driven model, and lowest in the WN-driven model (Figure 7). Most notably, the BG-driven model showed a prominent peak in causality in the high-beta/low-gamma band (20–35 Hz). This peak was almost entirely absent in the PD-driven model; for example, peak causality from layer 4 to layer 5 in this frequency range was only half that of the BG-driven model (0.23 and 0.45 for PD- and BG-driven models, respectively), even though these models had similar spectral power (Figure 6A). As shown in Figure 7, similar results were seen in other layer pairs (e.g., 4 → 2/3, 2/3 → 5, and 6 → 2/3).

## 4. Discussion

We have explored the effects of driving a spiking network model with several different types of input, including those corresponding to the healthy brain and to PD. Many of the differences between the healthy and PD models accord with prior experimental findings. For example, we found a modest but consistent reduction in firing rates of cortical neurons in PD. Although there are no direct studies of cortical firing rates during PD in humans, several indirect measures from functional imaging suggest such a decrease (Jenkins et al., 2004; Monchi et al., 2004, 2007). We also found a shift toward lower LFP frequencies, a finding consistent with PD electroencephalography (Soikkeli et al., 1991; Bosboom et al., 2006; Stoffers et al., 2007). We found increased synchrony between neurons in our PD model, as measured by population burst size and probability; increased synchrony among basal ganglia neurons is a commonly reported finding in PD (Raz et al., 1996), and increased synchrony among cortical neurons has also been reported (Goldberg et al., 2002).

Our major finding was the loss of Granger causality between cortical layers in the high-beta/low-gamma band. The Granger causality for the PD-driven model was more similar to the TC- and WN-driven models than to the BG-driven model, suggesting that the dynamical properties of the basal ganglia that facilitate cortical information flow are almost entirely lost in PD. The frequency range of this disrupted information flow is thought to be crucial for encoding motor commands, especially limb movements (Van Der Werf et al., 2008; Muthukumaraswamy, 2010). Gamma has also been implicated in many cognitive processes (Fries et al., 2007), including the perceptual binding underlying sensorimotor coordination (Lee et al., 2003) and consciousness (Llinas et al., 1998). Hence, our observation of disrupted causality might also partially account for some of the cognitive symptoms of PD, including bradyphrenia and planning deficits (Morris et al., 1988; Chaudhuri and Schapira, 2009).

The fact that Granger causality was disrupted in the PD-driven model (Figure 7) while the power spectrum was nearly unchanged in the same frequency band (Figure 6A) shows that the changed input drive has reorganized the dynamics of the network in complex ways. Since the GPi does not project directly to the cortex, these changes are entirely mediated by the thalamus; indeed, thalamic lesions alone are sufficient for producing parkinsonian symptoms in rats (Oehrn et al., 2007). Since the thalamus projects differentially to the different layers of the cortex, a major change in thalamic input is sufficient explanation for why the causality would shift so dramatically. Specifically, the thalamus normally projects strongly to layer 4; the peak in causality at 20–35 Hz is consistent with thalamic modulation by the GPi. In PD, inhibition to the thalamus is increased, which results in weaker drive to the cortex and thus a loss of information flow. Our findings suggest that therapeutic interventions, such as deep brain stimulation (Deuschl et al., 2006), may be more effective if they restore both the dynamics and the tonic level of activity of the GPi, rather than just the latter.

Several of our findings are qualitatively consistent with experimental results pointing to a loss of complexity in EEG time series from patients with a variety of cognitive disorders, including PD (Stam et al., 1994, 1995; Vaillancourt and Newell, 2002). For example, in the healthy model, the slope of the Fano factor increases roughly linearly on time scales from 1 ms to 10 s, indicating dynamical structure across a wide range of time scales (Figure 5D). This result can be seen qualitatively in the LFP time series of the healthy model, which appeared to show meaningful structure over a broader range of time scales than any of the other models (Figure 5B). We speculate that these properties may reflect the number of possible states that the network can assume, which may in turn be related to the number of different motor programs that can be implemented by the network. This principle is closely related to the concept of ϕ, defined as “the repertoire of causal states available to a system as a whole” (Balduzzi and Tononi, 2008). While ϕ cannot be easily computed for moderately large networks such as ours, we expect that it will be manifested in terms of the network's ability to perform real motor tasks—a topic we will explore in future work. Specifically, we predict that the BG-driven model will perform better on simulated reaching tasks than the WN-, TC-, or PD-driven models.

Beta-band activity (15–30 Hz) was predominantly generated by the thalamic and inhibitory cortical neuronal populations in our model (Figure 4B, top panel), in agreement with previous experimental and modeling studies (Brown and Williams, 2005; Hahn and McIntyre, 2010). Most empirical studies of beta activity in PD have focused on the basal ganglia nuclei, with increased power in the STN being a commonly reported finding (Brown and Williams, 2005; Kühn et al., 2005; Weinberger et al., 2006). In our model, we found that beta power in the STN was indeed enhanced in PD (Figure 4B, middle and bottom panels), which may reflect an idling or antikinetic state (Brown and Williams, 2005; Engel and Fries, 2010).

### 4.1. Limitations

Several experimentally observed features of PD, such as increased coherence among neurons in the STN, can only be explicitly represented using a neuronal network model of the basal ganglia (Terman et al., 2002)—a major benefit of that modeling approach. However, it is not known whether these phenomena are causally linked to parkinsonian symptoms. Hence, in the present context, the benefits of using a neural field model for the basal ganglia outweigh the drawbacks of this approach. In future, a spiking network model of the basal ganglia would be desirable in order to account for these and other phenomena, such as reinforcement learning. An explicit representation of dopamine in such a mode—rather than the implicit representation used here—would also allow the effects of pharmacological interventions to be modeled directly.

Due to the eloquence of the motor system, movement disorders are the most obvious symptoms of PD. Yet the pathophysiology of the disease is widespread; even the retina is affected (Hajee et al., 2009). We stress that the spiking network model used here was designed as a model of association cortex, not primary motor cortex; for example, our model includes layer 4 cells, which are absent from the latter. However, since the thalamus and striatum have broad projections to the cortex, we expect the dynamical and information-theoretic changes in PD (such as increased synchrony and reduced complexity) to extend to motor areas as well. In the future, we will explore the effects of PD in a model of primary motor cortex controlling a virtual arm (Chadderdon et al., 2012), with the aim of directly demonstrating classical parkinsonian motor symptoms. By incorporating sensory feedback into this model, the white noise that was used to drive the neural field component can be replaced with more realistic input, thereby addressing another obvious limitation of the method used here.

### 4.2. Multiscale dynamics in a composite model

To our knowledge, this work represents the first composite spiking network/neural field model of the brain. This is a multiscale model that spans spatial scales from 10 μm to 30 cm and temporal scales from 1 ms to tens of seconds. The composite method provides a way of linking two types of models that provide access to different spatial scales—a network model than spans scales from individual neurons (10 μm) to a cortical column (600 μm), and a field model encompassing the whole diencephalon (30 cm). Temporally, both network and field models are valid over many orders of magnitude (approximately 10^−3^−10^4^ s).

The mechanism used here to couple the field and network models is just one of several alternatives (Wilson et al., 2012). In the present case, the coupling was unidirectional; the network model did not affect the dynamics of the field model. While this can be easily justified in terms of the effective size of each model, an alternative approach generates the neural field based on the dynamics of the network model, using the new neuron-in-cell approach of Robinson and Kim (2012). Because spiking network models are still limited in their capacity to generate accurate dynamics on a large scale, this approach cannot yet be used in place of neural field models. However, this may change if scientific advances and improved computing facilities enable the development of larger and more realistic spiking network models.

Many spiking network models that are too small to show self-sustaining activity are driven by white noise (Hill and Tononi, 2005; Vogels and Abbott, 2005; Oswald et al., 2009; McDonnell et al., 2011; Volman et al., 2011; Kerr et al., 2012; Muller and Destexhe, 2012; Vijayan and Kopell, 2012). Here we demonstrated that using physiologically realistic input instead of white noise has a major impact on multiple measures of network activity, including power spectra, spiking variability, burst probability, and Granger causality. Thus, white-noise-driven spiking network models are an abstraction away from the physiological environment, and should perhaps be considered as being analogous to artificially driven slice preparations rather than *in vivo* activity.

## Conflict of interest statement

The authors declare that the research was conducted in the absence of any commercial or financial relationships that could be construed as a potential conflict of interest.
